# Mechanical Running Power and Energy Expenditure in Uphill and Downhill Running

**DOI:** 10.3390/sports13090294

**Published:** 2025-09-01

**Authors:** Fabrizio Gravina-Cognetti, Diego Chaverri, Antoni Planas, Jordi Montraveta, Marta Carrasco-Marginet, Silvia Puigarnau, Javier Espasa-Labrador, Xavier Iglesias

**Affiliations:** 1 INEFC-Barcelona Sport Sciences Research Group (GRCEIB), National Institute of Physical Education of Catalonia (INEFC), University of Barcelona, 08038 Barcelona, Spain; fgravina@gencat.cat (F.G.-C.); dchaverri@gencat.cat (D.C.); mcarrascom@gencat.cat (M.C.-M.); spuigarnau@gencat.cat (S.P.); xiglesias@gencat.cat (X.I.); 2 Faculty of Health Sciences, University of Valladolid (UVa), 47002 Valladolid, Spain; 3 Human Movement Research Group (GRMH), National Institute of Physical Education of Catalonia (INEFC), University of Lleida, 25192 Lleida, Spain; aplanas@gencat.cat

**Keywords:** trail running, energy expenditure, metabolism, athletic performance, nutrition, races

## Abstract

Trail running involves constant changes in terrain and slope, complicating the accurate assessment of energy expenditure during performance. This study aimed to examine the relationship between running power output (RPO), oxygen consumption (VO_2_), carbon dioxide production (VCO_2_), and energy expenditure per minute (EE_min_) across positive and negative slopes in trained trail runners under standardized laboratory conditions. Fifteen male trail runners performed five randomized 5 min treadmill runs at 70% of VO_2_ maximal speed on −7%, −5%, 0%, +5%, and +7% slopes. VO_2_, VCO_2_, EE_min_, respiratory exchange ratio (RQ), heart rate (HR), and RPO were recorded. Statistical analysis included Shapiro–Wilk tests for normality, repeated-measures ANOVA to compare variables across slopes, and Spearman or Pearson correlations between RPO and physiological variables. Moderate to strong positive correlations were found between RPO and VO_2_ (Rho = 0.80–0.84, *p* < 0.001) and between RPO and EE_min_ (Rho= 0.74–0.87, *p* < 0.01) across all conditions. These findings suggest that RPO measured via a wearable device may reflect changes in energy expenditure and supports the integration of wearable power metrics into training and nutritional strategies for trail running. However, further studies in female athletes, outdoor settings, extreme slopes, and altitude conditions are needed to confirm the generalizability of these results.

## 1. Introduction

Trail running races have significantly grown in popularity among runners driven by its unique environmental challenges, including altitude changes, uneven terrain, constant elevation shifts, and steep slopes, covering a wide range of distances, from shorter uphill vertical kilometer to long-distance ultra races [1]. These factors have generated interest within the scientific community, as these races push human physiology to its limits [2]. The variations in terrain and distance characteristic of trail running races demand a comprehensive understanding of the physiological responses for optimizing both performance and health [3,4].

In mountain running, for better performance runners often alternate strategically between walking and running based on gradient and terrain type [5]. This challenge makes it difficult to accurately monitor physical demands using metrics like average pace (time per kilometer) or speed variables commonly employed in road races [6] that fail to account for the complexities of terrain in mountain running. As a result, coaches, nutritionists, athletes, and researchers often lack reliable data for quantifying performance and energetic demand, limiting their ability to optimize training, recovery, and race preparation strategies [7]. This mechanical effort to accelerate, maintain speed, or brake is supported by the body’s energy expenditure (EE) obtained through the different metabolic pathways. EE is a key factor in endurance sports, as maintaining an appropriate energy balance is crucial for performance optimization and athlete health [8]. Negative energy balance, where energy output exceeds intake, can lead to adverse outcomes such as muscle mass loss, increased fatigue, and a decline in overall performance [9].

Currently, scientific evidence suggests that EE can be monitored in real time through various methods. The most popular monitoring method for estimating EE is cardiac activity, due to its strong correlation with oxygen consumption [10]. In endurance sports, heart rate (HR) is often used as an indicator due to its linear relationship with oxygen uptake (VO_2_) [11]. However, this relationship breaks down at extreme intensities, during intermittent efforts, or under the influence of factors like emotions, posture, and environmental conditions [12]. Prolonged exercise, particularly in warm environments, often induces cardiovascular drift, an upward shift in HR accompanied by a reduction in stroke volume, exacerbated by dehydration and thermal strain. These responses are associated with reduced plasma volume, impaired venous return, and increased sympathetic activation and can occur independently of any change in oxygen uptake [13]. Sleep restriction or deprivation can also elevate submaximal HR through increased sympathetic drive and altered thermoregulation, even when oxygen uptake remains unchanged [14]. Both conditions are commonly encountered in trail running, either due to early race start times, prolonged ultradistance events, or sustained efforts in warm environments and can lead to a decoupling of HR from VO_2_, reducing the reliability of HR as indicator of energetic demand. Alternatively, indirect calorimetry is considered the gold standard for accurately measuring EE and physiological responses [15], yet its use is limited in the laboratory setting. Consequently, other systems have been highlighted in the scientific literature as possible ways for solving the issue and try to estimate this data. One of them is the possibility of using Global Navigation Satellite Systems watches, very frequently used by mountain runners. This method provides speed and distance, two variables proposed for estimating EE; however, their accuracy in assessing EE is often questioned [16]. Thus, accelerometers have been proposed as a potentially reliable alternative [17]. Although early models, limited to single-axis measurements, presented notable challenges in reliability [18], recent advancements including three-dimensional axis capabilities. The triaxial accelerometer, with its metrics of mechanical power, stride length, and ground contact time, can detect changes in running technique [19], attributable to terrain characteristics. Also, this sensor enhances its ability to capture kinematic data if they are combined with other sensors such as gyroscopes and magnetometers [20]. This new and accurate approach brings to practitioners a novel method for monitoring training load in the trail running population, especially if we take into account the complexities of this discipline characterized by varying terrain, steep gradients, and alternating movement strategies [21]. In this context, the ability to obtain real-time data through wearable technology represents a significant advancement in training load monitoring [22]. Despite the well-established understanding of the theoretical models of metabolic cost and mechanical work during level and uphill running [23,24], the relationship between mechanical running power and energy expenditure across different inclines remains unexplored. While previous studies have demonstrated a relationship between power mechanical and metabolic parameters [25], none have directly analyzed whether instantaneous mechanical power can reliably predict energy expenditure across a full range of inclinations, including negative slopes. Understanding this relationship is essential for optimizing training load management and nutritional strategies in endurance athletes, particularly in mountain and trail running disciplines. 

The aim of this study was to explore the relationship between oxygen uptake, carbon dioxide, and mechanical running power at different slopes.

Additionally, this study sought to determine the relationship between EE per minute and instantaneous running power output (RPO) readings at varying slopes, both positive and negative.

## 2. Materials and Methods

### 2.1. Participants

A convenience [26] sample of 15 high-endurance-trained [27] mountain runners, males (*n* = 15), participated in this trial (age 37.27 ± 6.55 years; body weight 70.89 ± 7.05 kg; height 176.06 ± 5.96 cm; body mass index 22.85 ± 1.63 kg·m^−2^) (Table 1). The sample size was consistent with that of previous studies involving trail runners [28]. The inclusion criteria were to train at least 10 h per week, all participants had at least three years of mountain running experience and were injury-free for at least the preceding three months. Competitive level was assessed using the International Trail Running Association (ITRA) Performance Index, a globally recognized scoring system based on recent race performances that allows for standardized comparison between trail runners [29]. Intentions, procedures, as well as potential risks and benefits were communicated to the participants and were confirmed by signing an informed consent form according to the Declaration of Helsinki. This study was approved by the Committee for Clinical Investigations of the Sports Administration of Catalonia (020-CEICGC-2022).

**Figure 1:**
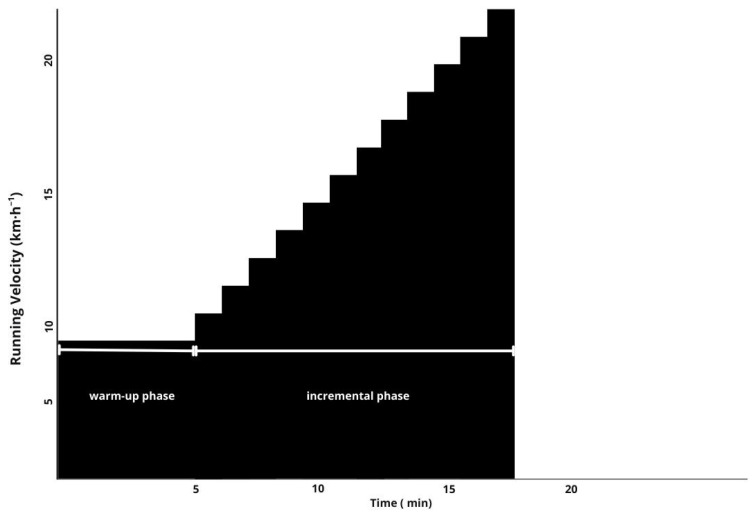
Schematic representation of the maximal oxygen uptake (VO_2max_) in the incremental treadmill test used in the present study. The protocol consisted of a 5 min warm-up phase at a constant speed of 8 km·h^−1^, followed by an incremental phase starting at 10 km·h^−1^ with speed increases of +1 km·h^−1^ every minute until volitional exhaustion. The participants average test duration (excluding the warm-up) was 9′37″, SD ± 1′ 10″.

Participants’ height (Holtain^®^ stadiometer, Holtain Limited^©^, Crosswell, UK) and body mass were measured (Seca 220^®^ scale, Seca Corp^©^, Hamburg, Germany) before starting running protocols. The first running test was aimed to obtain maximal peak values of physiological variables of interest by performing an incremental maximal protocol on a motorized treadmill (Cosmos HP, Nussdorf-Traunstein, Germany). Among these, the maximal oxygen consumption (VO_2max_) (Cosmed K5^®^, Cosmed SRL, Rome, Italy), maximal speed at VO_2_max (vVO_2_max), and maximal heart rate (HRmax) (Polar H10^®^, Polar Electro Oy, Kempele, Finland; firmware v3.2.0, software v7.15) were determined. Also, participants were equipped with an inertial movement unit, Stryd Footpod (Stryd, Boulder, CO, USA; firmware v2.1.15, software v7.8), which incorporates a triaxial accelerometer, a gyroscope, and a barometer into a compact shoe-mounted chip, allowing the researchers to obtain the mechanical power, expressed as running power output RPO applied on the treadmill during the test [30]. Specifically (Figure 1), after a 5 min warm-up at 8.0 km/h, the incremental test began at 10.0 km/h and increased by 1 km/h every minute until volitional exhaustion [31]. The average test duration (excluding the warm-up) was 9 min and 37 s, with an SD of ±1 min and 10 s.

**Figure 2:**
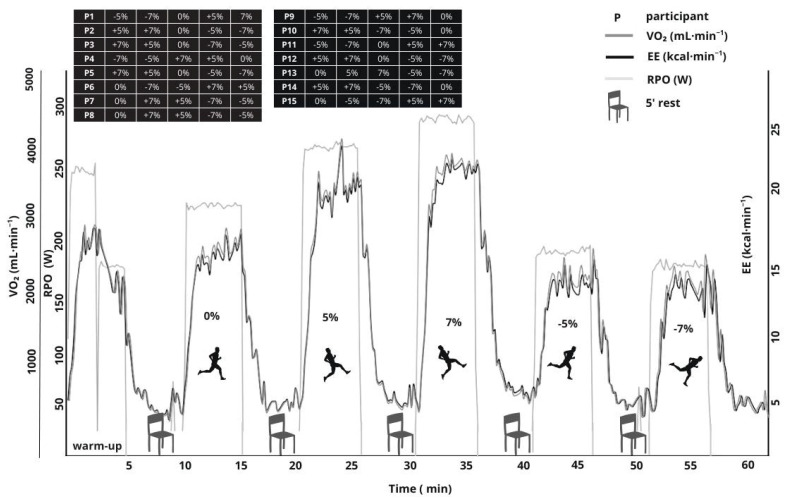
Illustrative example from one participant (P13). VO_2_ (mL·min^−1^), EE (kcal·min^−1^), and RPO (W) during treadmill running at five slopes (0%, ±5%, and ±7%) at constant speed (m·s^−1^). Each trial lasted 5 min with a 5 min passive recovery. Trials were performed consecutively in a single session; slope order was randomized.

A week later, all participants were called to the laboratory again to do the experimental protocol (Figure 2): a running test with different slopes [32,33]. This protocol consisted of running five series of five minutes at 70% of the speed associated with each participant’s maximal oxygen uptake which was selected as the standardized speed for all subsequent trials. This standardized speed and slopes were selected to elicit measurable physiological responses across slope conditions while ensuring submaximal intensity below the second ventilatory threshold [34], thus preserving the metabolic validity of the protocol. Varying slope conditions (−7%, −5%, 0%, +5%, and +7%) were administered in five separate trials of 5 min each, with a 5 min passive seated recovery between trials. The order of the slopes was randomized for each participant. During each set of the test, VO_2_, carbon dioxide output (VCO_2_), and respiratory exchange ratio (RQ) were monitored using the Cosmed K5^®^ portable system for recording breath-by-breath gas exchange measurements (Cosmed SRL, Rome, Italy). From gas exchange data, energy expenditure per minute (EE_min_) was calculated using Weir’s equation [35]. HR was assessed by the Polar H10^®^ (Polar Electro Oy, Kempele, Finland). Rating of perceived exertion (RPE) was assessed at the end of each set using the Borg CR10 scale following standardized procedures [36].

Running power output was assessed using the Stryd Footpod (Stryd, Boulder, CO, USA) which has been previously validated for running power estimation under controlled conditions [25] and managed via the Stryd mobile application (firmware v2.1.15, software v7.8), with manual input of slope prior to each effort. Data were uploaded to the Stryd Power Center platform [37], exported in FIT format, and converted to .csv files using Golden Cheetah (version 3.4), a free-license software, for further analysis in Microsoft Excel^®^ (2016; Microsoft Corp., Redmond, WA, USA).

While all these data were monitored during the total duration of each set, mean values from minutes two to four of each set were analyzed to better reflect steady-state physiological responses. All running tests were performed under the same ambient laboratory’s conditions (temperature 21.7 ± 1.2 °C; humidity 78.0 ± 7.4%).

### 2.2. Statistical Analysis

Descriptive statistics (mean ± standard deviation) were computed for all variables considering two levels of analysis: (i) the entire sample, and (ii) subgroups clustered according to slope conditions. The distribution of the data was first verified using the Shapiro–Wilk test. From this moment, either parametric or nonparametric procedures were subsequently applied. Associations between VO_2_ and VCO_2_ with PO and EE_min_ across multiple slope conditions (+7%, +5%, 0%, −5%, and −7%) were examined by correlation analysis. Depending on the distribution, Pearson’s r or Spearman’s rank correlation coefficient was used. Correlation strength was interpreted following Cohen’s [38] thresholds: small (0–0.30), moderate (0.31–0.49), large (0.50–0.69), very large (0.70–0.89), and nearly perfect (≥0.91). Additionally, differences analyses were conducted to compare differences across the five slope conditions (+7%, +5%, 0%, −5%, and −7%) and one-way analysis of variance was employed for normally distributed variables, while the Kruskal–Wallis test was applied when the assumption of normality was violated [39].

For variables that followed a normal distribution, homogeneity of variances was assessed with Levene’s test. When the assumption of equal variances was confirmed, Fisher’s was applied, whereas Welch’s was used when variances were unequal [39]. Post hoc comparisons were then performed to identify specific group differences, using Tukey’s for equal variances and the Games–Howell procedure for unequal variances. For variables showing significant group differences in the Kruskal–Wallis test, pairwise comparisons were conducted using the Dwass–Steel–Critchlow–Fligner procedure [39].

Statistical significance was set at *p* < 0.05. Data organization was performed using Microsoft Excel, and all statistical analyses were conducted using Jamovi (version 2.3; The Jamovi Project, 2023) [39]. Most metabolic variables (VO_2_, RPO, and EE_min_) exhibited a non-normal distribution (*p* < 0.05) for all slope conditions, necessitating the use of Spearman’s rank correlation (Rho). However, RQ showed normality in some conditions (0% and +5%), allowing Pearson’s correlation (*r*) to be used when applicable. Post hoc power for correlation tests was computed (two-tailed; α = 0.05), using Spearman’s (Rho) for power estimation, with n = 15 per slope. The Kruskal–Wallis test was conducted to compare differences across the five slope conditions (+7%, +5%, 0%, −5%, and −7%). The results of the Kruskal–Wallis test indicated that there were significant differences in the variables across the different slopes (*p* < 0.001), confirming the need for nonparametric analysis. Following this, Games–Howell post hoc tests were applied to examine specific differences between the slope conditions. These tests revealed significant differences between several pairwise comparisons (e.g., between +7% and −7%, between -% and 0%, and between 0% and +7%, all with *p* < 0.05), highlighting the impact of different slopes on metabolic variables.

## 3. Results

**Figure 3:**
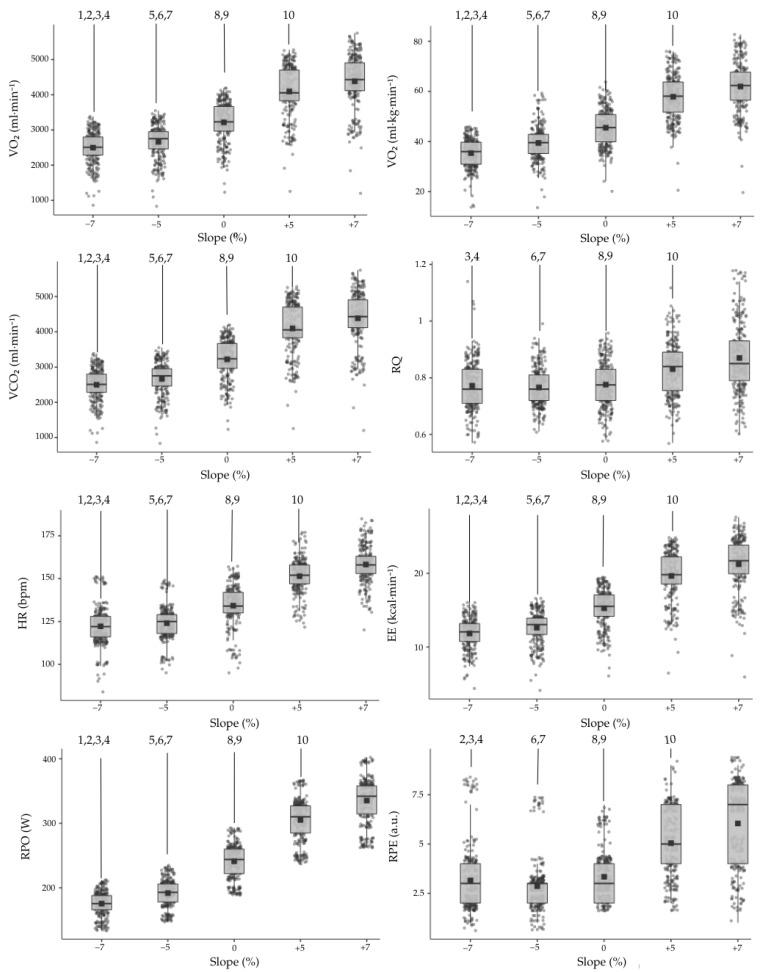
Mean (±SD) values of VO_2_ (mL·min^−1^), VCO_2_ (mL·min^−1^), RQ, HR (bpm), EE (kcal·min^−1^), RPO (W), and RPE during treadmill running at five slopes (−7%, −5%, 0%, +5%, and +7%) at a constant speed of 70% vVO_2_max. Each trial lasted 5 min, with data averaged from minutes 2 to 4. Numbers (1–10) indicate statistically significant pairwise differences between slopes (*p* < 0.05, see legend).

The descriptive analyses of each variable according to the different slopes are summarized in Table 2, while the differences are shown in Figure 3.

### 3.1. Descriptive and Difference Analysis

The mean of all analyzed parameters showed an increasing trend from the series with the steepest negative slope (−7%) to those with the steepest positive slope (+7%), except for RQ and RPE which showed higher values for the −7% slope to the −5% slope. This phenomenon was also observed when evaluating the differences among the various variables and slopes, identifying statistically significant differences across all groups except for RQ and RPE. In the case of RQ, differences were identified only for −7% vs. 5%, −7% vs. 7%, and −5% vs. 0%. On the other hand, RPE did not show a difference between the −7% and −5% groups. Lastly, while all differences were significant with a *p*-value < 0.001, the analysis of RQ between +5% and +7% was *p* = 0.008, HR showed *p* = 0.023 between −7% and −5%, and RPE between −7% and 0% groups also showed a large p value.

1: significant difference between the −7% and −5% slope; 2: significant differences between the −7% and 0% slope; 3: significant differences between the −7% and 5% slope; 4: significant difference between the −7% and 7% slope; 5: significant difference between the −5% and 0% slope; 6: significant difference between the −5% and 5% slope; 7: significant difference between the −5% and 7% slope; 8: significant difference between the 0% and 5% slope; 9: significant difference between the 0% and 7% slope; 10: significant difference between the 5% and 7% slope. a.u.: arbitrary unit; VO_2_: oxygen uptake; VCO_2_: carbon dioxide; RQ: respiratory quotient; HR: heart rate; EE_min_: energy expenditure minute; RPO _(W)_: running power output; RPE: rate of perceived exertion.

### 3.2. Correlation Analysis

Significant positive correlations were found among VO_2_, VCO_2_, EE_min_, and RPO, both when analyzing all slopes collectively and in the subgroup analyses (Table 3). In the full dataset analysis, the highest correlation was observed between VO_2_ and EE_min_ (Rho = 0.997, *p* < 0.001). This same pattern was evident in the subgroup analyses (Rho = 0.968–0.994, *p* < 0.001), with the strongest value recorded for the 7% slope. Additional near perfect correlations were also identified. Specifically, for the full dataset, near-perfect correlations were noted for VCO_2_ and EE·min^−1^ (Rho = 0.953, *p* < 0.001), VO_2_ and VCO_2_ (Rho = 0.929, *p* < 0.001), RPO and EE·min^−1^ (Rho = 0.916, *p* < 0.001), VCO_2_ and RPO (Rho = 0.910, *p* < 0.001), and VO_2_ and RPO (Rho = 0.904, *p* < 0.001). However, this level of correlation was not observed when each slope was analyzed separately, where the correlations ranged from large to very large. Post hoc power by slope (two-tailed; α = 0.05) was 0.83–0.99 for RPO–VO_2_, 0.92–0.99 for RPO–EE_min_, and 0.52–0.87 for RPO–VCO_2_.

Regarding the correlations between VO_2_ and VCO_2_, the lowest values were observed at the +7% slope (Rho = 0.421, *p* = 0.119) reaching their highest at −5% (Rho = 0.675, *p* < 0.5). VO_2_ demonstrated a correlation with RPO in all subgroup analyses, with the highest value at 5% (Rho = 0.836, *p* < 0.001) and the lowest at 7% (Rho = 0.689, *p* < 0.001). Correlations between VCO_2_ and RPO were comparatively lower, although all were classified as large and very large. The lowest value was recorded at −7% (Rho = 0.525, *p* < 0.05), while slightly higher correlations were found for the −5%, 07%, 5%, and +5% slopes (Rho = 0.546, 0.675, 0.689, and 0.711, respectively; *p* < 0.01). Finally, the correlation between RPO and EE_min_ varied depending on the dataset under analysis: it was near perfect when all data were considered together (Rho = 0.916, *p* < 0.001) and very large for the −7%, 0% (Rho = 0.739; *p* < 0.01), −5%, +5%, and +7% slopes (Rho = 0.789, 0.871, and 0.779 respectively; *p* < 0.001).

## 4. Discussion

This study aimed to investigate the relationship between mechanical running power and energy expenditure in trained trail runners across a range of positive and negative slopes. A dual analytical approach was adopted combining all data to assess general trends and analyzing each slope individually to better understand both the global mechanical and metabolic relationship and the slope-specific athlete responses. The results of this analysis revealed a progressive increase in all primary metabolic and mechanical variables, including VO_2_, VCO_2_, EE_min_, HR, and RPO, from –7% to +7%, while running speed was held constant. These findings are consistent with prior research showing that metabolic costs increase with mechanical demand as slope increases [40,41]. When data were pooled across all participants and gradients, nearly perfect associations emerged between mechanical running power and key metabolic variables, aligning with previous studies which reported a strong association between power output measured by Stryd wearable devices and oxygen consumption during running at different slopes [25]. Conversely, when the data were examined separately by slope condition, the strength of the correlation between mechanical running power and metabolic demand was reduced from large to very large. These findings are discussed in detail below and contextualized on the existing scientific literature.

While speed has been widely recognized in the scientific literature as the predominant intensity variable for training prescription and performance evaluation [6], in trail running understanding the demands of intensity requires its consideration alongside other parameters, with slope being a fundamental factor [24]. The findings of this study suggest that, at a constant running speed, trail runners progressively increase their metabolic demand as slope increases. VO_2_ was lowest at the –7% gradient (2498.9 ± 414.8 mL·min^−1^) and highest at the 7% gradient (4387.2 ± 732.0 mL·min^−1^), necessarily accompanied by a rise in VCO_2_ (from 1904.9 ± 299.4 mL·min^−1^ to 3765.7 ± 593.0 mL·min^−1^). The relationship between these parameters, reflected by the RQ, increased from 77± 0.08 to 87 ± 0.10, corresponding to an approximate 80.1% rise in EE. This pattern was statistically significant across nearly all slope comparisons. The increase in VO_2_ during uphill running can be attributed to the elevated metabolic demand required to vertically displace the body’s mass against gravity [41], as well as the additional muscular effort needed to propel the limbs and maintain postural stability throughout the ascent [42]. When transitioning from level to uphill running, the mechanical challenge imposed by gravity necessitates a substantial increase in concentric contractions [43], particularly through flexor and extensor muscles, as the slope increased [40]. At a constant speed, Saito et al. [44] reported significantly higher activation in the vastus medialis (*p* = 0.003), vastus intermedius (*p* = 0.004), biceps femoris (*p* = 0.004), and medial gastrocnemius (*p* = 0.017) when running at a 10% incline compared to level ground. Similarly, Wall-Scheffler et al. [42] observed a progressive increase in the activation of the biceps femoris, vastus lateralis, gluteus maximus, and gluteus medius as the slope increased from 0% to 20%, particularly during the stance and propulsive phases. These neuromuscular adaptations support the elevated mechanical and metabolic demands observed under steeper gradient conditions, as reflected by a significant rise in the RQ, as reported in the present study.

The results are physiologically coherent when considering VCO_2_ during exercise, since during the uphill conditions VCO_2_ increased by approximately 52.2% from 0% to +7%. Notably, within the upper gradient segment (from +5% to +7%) VCO_2_ showed a further progressive rise of 12.1%, which was nearly twice the increase observed for VO_2_ (6.9%) in the same slope range. This disproportionate rise may reflect not only elevated oxidative metabolism but also a greater contribution of acid–base regulation mechanisms under steeper conditions. VCO_2_ not only reflects metabolic load under aerobic conditions but also captures the systemic response to acid–base pH homeostasis regulation by elevated exercise intensity [45]. A significant portion of the VCO_2_ increase under high-intensity conditions comes from bicarbonate buffering of lactate-derived hydrogen ions [46]. Although participant variability could partially influence individual responses, the consistent pattern of VCO_2_ increase may reflect a shift in substrate use and acid–base regulation, particularly in runners nearing their second ventilatory threshold [34].

All these findings underscore that even minor variations in slope, when running speed is held constant, generate a substantial change in metabolic demand. This phenomenon is particularly relevant for pacing and intensity management in prolonged mountain races, where slope-specific strategies may help preserve energy stores, influence substrate utilization [47], and optimize performance during sustained uphill efforts.

In this context, RPO showed a strong parallelism with physiological parameters, increasing from 175.6 ± 18.7 W at −7% to 335.0 ± 35.5 W at +7%. All slope-to-slope differences were statistically significant, with higher magnitude between 0% and +5% (+63.6 W) and between –5% and 0% (+49.8 W).

A significant positive correlation (Rho = 0.689–0.836, *p* < 0.01) was observed between RPO and VO_2_ across all slope conditions examined (as shown in Table 3). At ground level, the correlation between VO_2_ and RPO was very large (Rho = 0.805, *p* < 0.001). This alignment reflects the fundamental principle [48] that, in level locomotion, VO_2_ is directly proportional to the amount of mechanical work performed [48]. Notably, the strength of the relationship between RPO and VO_2_ declined at the steepest gradients, with correlation coefficients of Rho= 0.732 at −7% (*p* < 0.01) and Rho = 0.689 at +7% with *p* < 0.01, respectively. Accelerometry directly indicates running load [22], but RPO alone may not capture key aspects of internal load, such as the neuromuscular demands of muscle contractions, which substantially contribute to the energy cost but are not always reflected in power output. This progressive decoupling between metabolic demand and mechanical power data could reflect the increasing complexity of muscular recruitment and energy pathways activated during steep uphill running [32]. This heightened mechanical demand activates bioenergetic pathways within muscle cells, accelerating substrate oxidation and electron flux through the mitochondrial respiratory chain to sustain the elevated adenosine triphosfate requirements [49]. It is plausible that, as the slope steepens, this recruitment gradually extends from predominantly oxidative type I fibers to a broader pool that includes type II fibers, which are characterized by higher strength production but lower efficiency [50] and anaerobic characteristics.

When comparing 0% slope to the steepest downhill condition (−7%) at the same speed, VO_2_ decreased by 22.5%, reflecting the energy saving effects of downhill running, a phenomenon described previously in the literature [32,51]. Negative slopes are generally associated with lower physiological and mechanical demands compared to level running, primarily due to the gravitational assistance that reduces the need to generate force for active propulsion; therefore, there is a lower internal demand [3]. In moderate downhill running, tendinous structures such as the Achilles tendon and plantar arch contribute substantially to propulsion through passive elastic recoil [52]. The stretch–shortening cycle plays a key role in the mechanical and energetic efficiency of downhill running. During the eccentric phase of ground contact, elastic structures can absorb mechanical energy that is partially released during push-off, reducing the need for metabolically costly concentric contractions [47]. The lower correlation values may reflect the biomechanical and muscular adjustments that occur during descent, an effect previously described by Snyder and colleagues [52]. This could indicate an elevated VCO_2_ production, potentially due to greater eccentric muscle activation [53]. Specifically, downhill running often relies more on eccentric muscle contractions and less on concentric strength production, which can slightly attenuate the rate at which VO_2_ increases relative to mechanical power [51]. Additionally, individual muscle activation during downhill running may be influenced by metabolic and mechanical demands, neuromuscular skill, and terrain-specific experience [45].

These factors together could help explain the weaker association between mechanical power and metabolic parameters at steeper negative slopes and may also help explain the progressive uncoupling observed in our study between VCO_2_ and RPO at higher intensities of negative slope (Rho= 0.525, *p* < 0.05) (Table 3). Complementing this finding, internal load indicators [54] assessed in this study such as RQ and RPE demonstrate deviation from linearity under moderate downhill conditions.

Although no statistically significant differences were observed between the −7% and −5% slope conditions, both RQ (0.77 ± 0.08 vs. 0.76 ± 0.06) and RPE (3.03 ± 1.78 vs. 2.60 ± 1.39) showed higher values at the steeper negative gradient suggesting increased internal load. While total metabolic demand, as indicated by VO_2_ and VCO_2_, decreases during downhill running, the neuromuscular system may require the activation of stabilizing musculature such as the gluteal, hamstrings, and core muscles to control acceleration and maintain postural balance [52]. Braking forces are known to become dominant at gradients steeper than approximately −10% (22); the metabolic shift observed at −7% may reflect a transitional phase rather than a condition of fully eccentric-dominant muscle activity. All these differences appear to reflect intraindividual variations in efficiency when confronting different inclines. In other words, each mountain runner seems to respond uniquely to the mechanical and physiological demands of each gradient, which in turn modulates the strength of the coupling between external load and metabolic cost.

An additional objective of this study was to evaluate the relationship between mechanical running power and energy expenditure among the different slopes. For this purpose, the Weir’s formula was used to estimate EE_min_ across a variety of inclines [35]. This equation combines two fundamental components, VO_2_ and carbon dioxide VCO_2_, weighted by their respective caloric contributions to energy metabolism, and this ratio provides insight into which energy substrates are being oxidized to sustain movement [35]. By incorporating both variables, the formula provides a comprehensive estimate of energetic exercise demand [55], expressed in kilocalories per minute, which could explain why, across all slope conditions, the associations between RPO and EE_min_ were stronger (see Table 3) than those observed between RPO and isolated metabolic variables such as VO_2_ or VCO_2_. Across all slope conditions, EE_min_ and RPO showed consistently very large correlations (Rho= 0.739, *p* < 0.01; Rho = 0.871, *p* < 0.001) between RPO and EE_min_ in level and moderate slopes. As widely reported in the scientific literature, the energy required for movement represents only a fraction of the total EE [41], and RPO could represent this part. According to Riddick R. and Kuo A. [56], the mechanical work performed by the muscles to support body weight and propel the body, including the vertical and horizontal oscillations of the center of mass, accounts for approximately 60% to 76% of the energetic cost of running. This conceptual alignment may help explain the very large correlations observed in our study between Stryd-derived RPO and EE_min_ across varying slope conditions. Recent evidence [46] indicates that inertial sensors positioned on the foot, such as Stryd, are capable of reliably measuring spatiotemporal gait parameters such as step frequency, ground contact time, and vertical oscillation. Considering that VO_2_ is proportional to the strength exerted by active muscles, and that inertial sensors are capable of measuring movement across three spatial axes [57], it is reasonable to assume that, during cyclic activities such as running, in submaximal conditions, continuous acceleration data [58] can be used to understand external load. The estimation of mechanical power in running integrates multiple biomechanical [59] and kinematic parameters, including ground contact time, cadence, stride length, vertical oscillation, limb stiffness, and horizontal velocity [19]. As these variables are closely linked to the mechanical demands of running, their continuous monitoring may help explain the very large correlations observed in our study between Stryd derived RPO and EE_min_ across varying slope conditions. Of note, at the steepest inclines tested, we observed a slight reduction in this association, decreasing from Rho = 0.871 at +5% to Rho = 0.779 at +7% %, *p* < 0.001, and from Rho= 0.789 at −5% to Rho = 0.739 at −7%, *p* < 0.01, which may suggest that the relationship between RPO and EE_min_ weakens as the slope magnitude increases. The reduced association between RPO and EE_min_ at steeper slopes may be partly attributed to device accuracy under extreme gradient conditions. Additionally, inter-individual differences in gradient-specific running technique may further contribute to the observed decoupling between EE_min_ and RPO at the steepest slopes.

Approximately three-quarters of total energy expenditure is dedicated to essential physiological processes, including vital cellular functions, thermogenesis, and internal work, and not to produce movement [41]. Moreover, the efficiency with which an individual transforms metabolic energy into mechanical work is influenced by multiple factors, such as muscle fiber composition, biomechanical strategies, and neuromuscular coordination [24]. The integration of RPO with metabolic parameters may offer valuable insight into running efficiency across different slopes, as it reflects the relationship between external mechanical load and the associated metabolic cost under varying gradient conditions. Additionally, energy expenditure is influenced by terrain characteristics, duration of effort, and environmental conditions such as temperature, altitude, and cold [5]. These factors, which are particularly prevalent in mountain races, add complexity to the accurate assessment of energy expenditure. In uphill running, for example, specific biomechanical adaptations in spatiotemporal gait metrics are induced, with increased step frequency and reduced step length, which reflect a strategy to maintain propulsion [40] accompanied by a reduced tendon capacity for elastic energy storage [52]. In response to these changes, individual specific adaptations may emerge, influencing the efficiency of energy transfer and potentially modifying total energy expenditure.

The findings of this study demonstrate that, although a relationship exists between energy expenditure and absolute power values, power alone cannot fully account for the amount of energy expended during exercise. Nevertheless, the significant relationships observed confirm that accelerometry could be used as a proxy for estimating energy expenditure at different moderate inclines in trail runners.

### 4.1. Limitations

This study presents several limitations that should be acknowledged. First, the restricted range of slopes tested, from −7% to +7%, may limit the generalizability of the findings, particularly regarding the relationship between mechanical power output and metabolic responses at steeper inclines or declines. A broader spectrum of gradients could have provided deeper insights into the strength and consistency of the relationship between mechanical power output and energy expenditure, particularly at more extreme inclines or declines where this coupling may change.

Second, the sample consisted of a relatively small and heterogeneous group of 15 experienced male trail runners, which, although relevant to the population of interest, introduces variability in age, sex, and physical characteristics that may affect the consistency of the results. Finally, mechanical power was assessed using a single commercially available inertial sensor (Stryd), and while its use has been validated in previous research, reliance on a single device limits the extrapolation of the results to other technologies or measurement systems.

### 4.2. Practical Considerations

To optimize the performance (training, nutrition, and recovery periodization) in trail running events, practitioners should consider the terrain-specific demands by examining the course profile in detail, identifying the distribution of inclines and declines, the distance and duration of each segment, and the athlete’s pace within those contexts. This approach allows for a more accurate estimation of energy requirements and facilitates the development of personalized nutritional strategies tailored to the unique energetic demands of each phase of the race. Therefore, it is crucial that nutrition professionals thoroughly understand the specific device utilized by the athlete when estimating energy expenditure. Accurate knowledge of the tool is essential for developing effective pre-race, during-race, and post-race nutritional strategies, as different devices may yield varying estimates of energy expenditure. This multi-variable approach may contribute to a closer alignment between power measurements and energetic demand.

In estimating energy expenditure in athletes, particularly during long distance events such as ultra marathons, these systems measure energy expenditure primarily during movement, by tracking body acceleration, but they do not account for energy consumption when the body is stationary, such as during aid station stops. Despite the assumption that no energy is expended during rest, the body continues to consume energy for processes like thermoregulation, digestion, and postural maintenance, even when not moving. In long-distance events, aid station stops can account for significant portions of total race time, and their duration may vary depending on the athlete’s strategy and fueling needs. Nutritionists must account for both active and inactive energy expenditure when developing fueling strategies for endurance events. Neglecting the energy consumed during stationary periods could result in inaccurate energy requirements and suboptimal nutrition plans.

## 5. Conclusions

The data observed in this study, including correlations between mechanical power output and energy expenditure, confirm that running power data measured by a wearable inertial device could serve as a useful proxy for assessing external load and caloric needs during exercise. However, the variability observed across different slopes and the influence of individual efficiency and biomechanical adaptations highlight the limitations of relying solely on power measurements.

These findings emphasize the need for an integrative approach that combines running power data with metabolic and biomechanical information, especially when addressing the complexities of trail running. Future research should aim to validate these methods in ecological conditions and explore how terrain variability, environmental factors, sex, and individual physiological responses affect the accuracy of energy expenditure estimations.

## Figures and Tables

**Table 1:** Anthropometrics and physiological characteristics of the participants.

Characteristic	Mean ± SD
Age _(years)_(years)	37.27 ± 6.55
ITRA (points)	676 ± 101
Weight (kg)	70.89 ± 7.05
Height (m)	176.06 ± 5.96
BMI (kg·m^−2^)	22.85 ± 1.63
vVO_2_max (m·s^−1^)	4.85 ± 0.44
70% vVO2max (m·s^−1^)	3.33 ± 0.31
HRmax (bpm)	171.47 ± 10.31
VO2max (mL·min^−1^)	4307.38 ± 487.71
VO2max (mL·kg^−1^·min^−1^)	61.04 ± 6.91
RPOmax (W)	368.07 ± 38.04
RPOmax (W·kg^−1^)	5.20 ± 0.32

Age (years); ITRA (points): International trail running association ranking; Weight (kg); Height (m); BMI (kg·m^−2^): body mass index; vVO_2_max (m·s^−1^): maximal speed at VO_2_max; 70% vV;O_2_max (m·s^−1^): running speed set at 70% of the velocity associated with VO_2_max; VO_2_max (mL·kg^−1^_−1_·min^−1^): maximal oxygen uptake relative to weight; VO_2_max (mL·min^−1^): maximal oxygen uptake; RPOmax: maximal running power output; RPOmax (W·kg^−1^): maximal running power output relative to weight.

**Table 2:** Descriptive (mean and deviation) results of the 3 min mean values by slope.

	Slope				
	−7%	−5%	0%	5%	7%
VO_2_ (mL·min^−1^)	2521.4 ± 397.3	2682.8 ± 425.4	3233.7 ± 525.4	4111.6 ± 662.4	4390.2 ± 694.2
VO_2_ (mL·kg·min^−1^)	35.66 ± 4.89	37.91 ± 5.15	45.75 ± 6.85	58.12 ± 8.15	62.09 ± 8.87
VCO_2_ (mL·min^−1^)	1919.1 ± 290.9	2031.9 ± 283.6	2478.0 ± 372.9	3360.6 ± 470.8	3772.2 ± 569.9
RQ	0.77 ± 0.08	0.76 ± 0.06	0.77 ± 0.08	0.82 ± 0.09	0.87 ± 0.11
HR (bpm)	122.5 ± 11.0	124.3 ± 9.7	134.3 ± 11.7	151.6 ± 8.8	158.5 ± 9.4
EE (kcal·min^−1^)	11.9 ± 1.8	12.7 ± 1.9	15.3 ± 2.4	19.7 ± 3.0	21.3 ± 3.1
RPO (W)	175.9 ± 18.9	192.0 ± 21.3	242.3 ± 26.9	305.7 ± 32.5	335.1 ± 35.7
RPE	3.03 ± 1.78	2.60 ± 1.39	3.07 ± 1.10	4.80 ± 1.57	6.33 ± 1.79

a.u.: arbitrary unit; VO2 (mL·min^−1^): oxygen uptake; VO_2_ (mL·kg·min^−1^): relative oxygen uptake; VCO_2_ (mL·min^−1^): carbon dioxide; RQ: respiratory quotient; HR (bpm): heart rate; EE (kcal·min^−1^): energy expenditure minute; RPO (W): running power output; RPE: rate of perceived exertion.

**Table 3:** Correlation analysis.

Data Analysis		VO_2(ml/min)_	VCO_2 (ml/min)_	RPO _(W)_
All data included	VO_2 (mL·min^−1^)_	-		
	VCO_2 (mL·min^−1^)_	0.929 ***	-	
	RPO _(W)_	0.904 ***	0.910 ***	-
	EE _(kcal·min^−1^)_	0.997 ***	0.953 ***	0.916 ***
−7% slope	VO_2 ((mL·min^−1^)_	-		
	VCO_2 (mL·min^−1^)_	0.611 *	-	
	RPO _(W)_	0.732 **	0.525 *	-
	EE _(kcal·min^−1^)_	0.996 ***	0.636 *	0.739 **
−5% slope	VO_2 (mL·min^−1^)_	-		
	VCO_2 (mL·min^−1^)_	0.675 **	-	
	RPO _(W)_	0.789 ***	0.621 ***	-
	EE _(kcal·min^−1^)_	0.989 ***	0.729 **	0.789 ***
0% slope	VO_2 (mL·min^−1^)_	-		
	VCO_2 (mL·min^−1^)_	0.646 *	-	
	RPO _(W)_	0.805 ***	0.675 **	-
	EE _(kcal·min^−1^)_	0.982 ***	0.736 **	0.739 **
5% slope	VO_2 (mL·min^−1^)_	-		
	VCO_2 (mL·min^−1^)_	0.536 *	-	
	RPO _(W)_	0.836 ***	0.689 **	-
	EE _(kcal·min^−1^)_	0.957 ***	0.686 **	0.871 ***
7% slope	VO_2 (mL·min^−1^)_	-		
	VCO_2 (mL·min^−1^)_	0.421	-	
	RPO _(W)_	0.689 **	0.711 **	-
	EE _(kcal·min^−1^)_	0.957 ***	0.596 *	0.779 ***

Values are Spearman’s correlation coefficients (Rho) * *p* < 0.05; ** *p* < 0.01; *** *p* < 0.001. VO_2 (mL·min^−1^)_: oxygen uptake; VCO_2 (mL·min^−1^)_: carbon dioxide; EE _(kcal·min^−1^)_: energy expenditure minute; RPO _(W)_: running power output.

## Data Availability

The data supporting the findings of this study are available from the corresponding author upon reasonable request, due to the sensitive nature of the individual level performance metrics and the need to protect participant confidentiality.

## Abbreviations

The following abbreviations are used in this manuscript:

VO_2_	Oxygen uptake
VCO_2_	Carbon dioxide output
RQ	Respiratory quotient
HR	Heart rate
EE	Energy expenditure
EE_min_	Energy expenditure per minute
RPO	Running power output
RPE	Rating of perceived exertion
BMI	Body mass index
vVO_2max_	Velocity at maximal oxygen uptake
VO_2max_	Maximal oxygen uptake
ITRA	International Trail Running Association

## References

[1] Scheer V. (2019). Participation Trends of Ultra Endurance Events. Sports Med. Arthrosc..

[2] de Waal S.J., Gomez-Ezeiza J., Venter R.E., Lamberts R.P. (2024). Physiological Indicators of Trail Running Performance: A Systematic Review. Int. J. Sports Physiol. Perform..

[3] Vernillo G., Giandolini M., Edwards W.B., Morin J.B., Samozino P., Horvais N., Millet G.Y. (2017). Biomechanics and Physiology of Uphill and Downhill Running. Sport. Med..

[4] Boshielo P.M., Jansen van Rensburg A., Viljoen C., Botha T., de Villiers C.E., Ramagole D., Seyani L., Janse van Rensburg D.C. (2024). Illness Is More Prevalent than Injury in Trail Runners Participating in a Mountainous Ultra Trail Race. Phys. Sportsmed..

[5] Vernillo G., Savoldelli A., Zignoli A., Skafidas S., Fornasiero A., Torre A.L., Bortolan L., Pellegrini B., Schena F. (2015). Energy Cost and Kinematics of Level, Uphill and Downhill Running: Fatigue-Induced Changes after a Mountain Ultramarathon. J. Sports Sci..

[6] Abbiss C.R., Laursen P. (2008). Describing and Understanding Pacing Strategies. Sport. Med..

[7] Zimmermann P., Müller N., Schöffl V., Ehrlich B., Moser O., Schöffl I. (2022). The Energetic Costs of Uphill Locomotion in Trail Running: Physiological Consequences Due to Uphill Locomotion Pattern—A Feasibility Study. Life.

[8] Burke L.M., Jones A.M., Jeukendrup A.E., Mooses M. (2019). Contemporary Nutrition Strategies to Optimize Performance in Distance Runners and Race Walkers. Int. J. Sport Nutr. Exerc. Metab..

[9] Clemente-Suárez V.J. (2015). Psychophysiological Response and Energy Balance during a 14-h Ultraendurance Mountain Running Event. Appl. Physiol. Nutr. Metab..

[10] Spurr G.B., Prentice A.M., Murgatroyd P.R., Goldberg G.R., Reina J.C., Christman N.T. (1988). Energy Expenditure from Minute-by-Minute Heart-Rate Recording: Comparison with Indirect Calorimetry. Am. J. Clin. Nutr..

[11] Booyens J., Hervey G.R. (1960). The Pulse Rate as a Means of Measuring Metabolic Rate in Man. Can. J. Biochem. Physiol..

[12] Achten J., Jeukendrup A.E. (2003). Heart Rate Monitoring: Applications and Limitations. Sport. Med..

[13] Périard J.D., Eijsvogels T.M.H., Daanen H.A.M. (2021). Exercise under Heat Stress: Thermoregulation, Hydration, Performance Implications, and Mitigation Strategies. Physiol. Rev..

[14] Fullagar H.H.K., Skorski S., Duffield R., Hammes D., Coutts A.J., Meyer T. (2015). Sleep and Athletic Performance: The Effects of Sleep Loss on Exercise Performance, and Physiological and Cognitive Responses to Exercise. Sport. Med..

[15] Leonard W.R. (2012). Laboratory and Field Methods for Measuring Human Energy Expenditure. Am. J. Hum. Biol..

[16] Hongu N., Orr B.J., Roe D.J., Reed R.G., Going S.B. (2013). Global Positioning System Watches for Estimating Energy Expenditure. J. Strength Cond. Res..

[17] Mejer G., Westerterp K.R., Koper H. (1989). Assessment of Energy Expenditure by Recording Heart Rate and Body Acceleration. Med. Sci. Sports Exerc..

[18] Murakami H., Kawakami R., Nakae S., Nakata Y., Ishikawa-Takata K., Tanaka S., Miyachi M. (2016). Accuracy of Wearable Devices for Estimating Total Energy Expenditure: Comparison with Metabolic Chamber and Doubly Labeled water Method. JAMA Intern. Med..

[19] García-Pinillos F., Roche-Seruendo L.E., Marcén-Cinca N., Marco-Contreras L.A., Latorre-Román P.A. (2021). Absolute Reliability and Concurrent Validity of the Stryd System for the Assessment of Running Stride Kinematics at Different Velocities. J. Strength Cond. Res..

[20] Perrotin N., Gardan N., Lesprillier A., Le Goff C., Seigneur J.M., Abdi E., Sanudo B., Taiar R. (2021). Biomechanics of Trail Running Performance: Quantification of Spatio-Temporal Parameters by Using Low Cost Sensors in Ecological Conditions. Appl. Sci..

[21] Navalta J.W., Montes J., Bodell N.G., Aguilar C.D., Radzak K., Manning J.W., Debeliso M. (2019). Reliability of Trail Walking and Running Tasks Using the Stryd Power Meter. Int. J. Sports Med..

[22] Van Hooren B., Goudsmit J., Restrepo J., Vos S. (2020). Real-Time Feedback by Wearables in Running: Current Approaches, Challenges and Suggestions for Improvements. J. Sports Sci..

[23] Margaria R., Cerretelli P., Aghemo P., Sassi G. (1963). Energy Cost of Running. J. Appl. Physiol..

[24] Minetti A.E., Moia C., Roi G.S., Susta D., Ferretti G. (2002). Energy Cost of Walking and Running at Extreme Uphill and Downhill Slopes. J. Appl. Physiol..

[25] Cerezuela-Espejo V., Hernández-Belmonte A., Courel-Ibáñez J., Conesa-Ros E., Mora-Rodríguez R., Pallarés J.G. (2021). Are We Ready to Measure Running Power? Repeatability and Concurrent Validity of Five Commercial Technologies. Eur. J. Sport Sci..

[26] Barnes K.R., Kilding A.E. (2015). Running Economy: Measurement, Norms, and Determining Factors. Sport. Med.-Open.

[27] McKay A.K.A., Stellingwerff T., Smith E.S., Martin D.T., Mujika I., Goosey-Tolfrey V.L., Sheppard J., Burke L.M. (2022). Defining Training and Performance Caliber: A Participant Classification Framework. Int. J. Sports Physiol. Perform..

[28] Björklund G., Swarén M., Born D.P., Stöggl T. (2019). Biomechanical Adaptations and Performance Indicators in Short Trail Running. Front. Physiol..

[29] International Trail Running Association. https://itra.run/Runners/Performance.

[30] Imbach F., Candau R., Chailan R., Perrey S. (2020). Validity of the Stryd Power Meter in Measuring Running Parameters at Submaximal Speeds. Sports.

[31] Noakes T.D., Myburgh K.H., Schall R. (1990). Peak Treadmill Running Velocity during the Vo2 Max Test Predicts Running Performance. J. Sports Sci..

[32] Padulo J., Powell D., Milia R., Ardigò L.P. (2013). A Paradigm of Uphill Running. PLoS ONE.

[33] Billat L.V., Koralsztein J.P. (1996). Significance of the Velocity at VO2max and Time to Exhaustion at This Velocity. Sports Med..

[34] Beaver W.L., Wasserman K., Whipp B.J. (1986). A New Method for Detecting Anaerobic Threshold by Gas Exchange. J. Appl. Physiol..

[35] Weir J.B.d.V. (1949). New Methods for Calculating Metabolic Rate with Special Reference to Protein Metabolism. J. Physiol..

[36] Foster C., Florhaug J.A., Franklin J., Gottschall L., Hrovatina L.A., Suzanne P., Doleshal P., Dodge C. (2001). A New Approach to Monitoring Exercise Training. J. Strength Cond. Res..

[37] Stryd Power Center. https://www.stryd.com/powercenter.

[38] Gusakov M.Y., Yakovenko T.V., Latysheva E.K., Zaval’naya G.I., Ovdak A.P. (1984). Effect of the Degree of Polymerization of Polycaproamide on the Strength of Technical Purpose Yarn. Fibre Chem..

[39] Lalanne C., Mesbah M. (2016). Measures of Association, Comparisons of Means and Proportions for Two Samples or More. Biostatistics and Computer-Based Analysis of Health Data Using Stata.

[40] Padulo J., Annino G., Smith L., Migliaccio G.M., Camino R., Tihanyi J., Dottavio S. (2012). Uphill Running at Iso-Efficiency Speed. Int. J. Sports Med..

[41] Margaria R. (1968). Positive and Negative Work Performances and Their Efficiencies in Human Locomotion. Int. Z. Angew. Physiol. Einschließlich Arbeitsphysiologie.

[42] Wall-Scheffler C.M., Chumanov E., Steudel-Numbers K., Heiderscheit B. (2010). Electromyography Activity across Gait and Incline: The Impact of Muscular Activity on Human Morphology. Am. J. Phys. Anthropol..

[43] Olesen H.L. (1992). Accumulated Oxygen Deficit Increases with Inclination of Uphill Running. J. Appl. Physiol..

[44] Saito A., Tomita A., Ando R., Watanabe K., Akima H. (2018). Muscle Synergies Are Consistent across Level and Uphill Treadmill Running. Sci. Rep..

[45] Breiner T.J., Ortiz A.L.R., Kram R. (2019). Level, Uphill and Downhill Running Economy Values Are Strongly Inter-Correlated. Eur. J. Appl. Physiol..

[46] Jaén-Carrillo D., Roche-Seruendo L.E., Cartón-Llorente A., Ramírez-Campillo R., García-Pinillos F. (2020). Mechanical Power in Endurance Running: A Scoping Review on Sensors for Power Output Estimation during Running. Sensors.

[47] Brooks G.A., Mercier J. (1994). Balance of Carbohydrate and Lipid Utilization during Exercise: The “crossover” Concept. J. Appl. Physiol..

[48] Cavagna G.A., Kaneko M. (1977). Mechanical Work and Efficiency in Level Walking and Running. J. Physiol..

[49] Murphy M.P. (2009). How Mitochondria Produce Reactive Oxygen Species. Biochem. J..

[50] Plotkin D.L., Roberts M.D., Haun C.T., Schoenfeld B.J. (2021). Muscle Fiber Type Transitions with Exercise Training: Shifting Perspectives. Sports.

[51] Bontemps B., Vercruyssen F., Gruet M., Louis J. (2020). Downhill Running: What Are The Effects and How Can We Adapt? A Narrative Review. Sport. Med..

[52] Snyder K.L., Kram R., Gottschall J.S. (2012). The Role of Elastic Energy Storage and Recovery in Downhill and Uphill Running. J. Exp. Biol..

[53] Chen T.C., Nosaka K., Tu J.H. (2007). Changes in Running Economy Following Downhill Running. J. Sports Sci..

[54] Halson S.L. (2014). Monitoring Training Load to Understand Fatigue in Athletes. Sport. Med..

[55] Hills A.P., Mokhtar N., Byrne N.M. (2014). Assessment of Physical Activity and Energy Expenditure: An Overview of Objective Measures. Front. Nutr..

[56] Riddick R.C., Kuo A.D. (2022). Mechanical Work Accounts for Most of the Energetic Cost in Human Running. Sci. Rep..

[57] Wilson R.P., Börger L., Holton M.D., Scantlebury D.M., Gómez-Laich A., Quintana F., Rosell F., Graf P.M., Williams H., Gunner R. (2020). Estimates for Energy Expenditure in Free-Living Animals Using Acceleration Proxies: A Reappraisal. J. Anim. Ecol..

[58] Sutton G.J., Botha J.A., Speakman J.R., Arnould J.P.Y. (2021). Validating Accelerometry-Derived Proxies of Energy Expenditure Using the Doubly Labelled Water Method in the Smallest Penguin Species. Biol. Open.

[59] Berzosa C., Comeras-Chueca C., Bascuas P.J., Gutiérrez H., Bataller-Cervero A.V. (2024). Assessing Trail Running Biomechanics: A Comparative Analysis of the Reliability of StrydTM and GARMINRP Wearable Devices. Sensors.

